# Supplementary material for: The 30-Day Economic Burden of Newly Diagnosed Complicated Urinary Tract Infections in Medicare Fee-for-Service Patients Who Resided in the Community
Source: Antibiotics (Basel). 2022 Apr 26;11(5):578. doi: 10.3390/antibiotics11050578 (PMC9137853; doi:10.3390/antibiotics11050578)
Supplement: Supplementary file 1 [file antibiotics-11-00578-s001.zip › antibiotics-1671893-supplementary.pdf]

Supplemental Table S1. Algorithm for Identifying Medicare Beneficiaries with cUTIs Based on International Classification of Diseases, Tenth Revision (ICD-10) Diagnostic Codes and Procedure Codes and Current Procedural Terminology (CPT) Codes

Group A		Group B		Group C	
Any code from this group qualifies as UTI<br>Presence on inpatient claim qualifies as cUTI		Any code from Group B qualifies as UTI<br>Group B code and Group C code on same day qualifies as cUTI			
**ICD-10 diagnosis**<br>• N10<br>• N110<br>• N118<br>• N12<br>• N151<br>• N159<br>• N16<br>• N2884<br>• N2885<br>• N2886	**ICD-10 diagnosis**<br>• N35111<br>• N35112<br>• N35113<br>• N35114<br>• N35119<br>• N3512<br>• T83510A<br>• T83511A<br>• T83512A<br>• T83518A	**ICD-10 diagnosis**<br>• N139<br>• N3000<br>• N3010<br>• N3020<br>• N3030<br>• N3040<br>• N3080<br>• N3090<br>• N3001<br>• N3011<br>• N3021<br>• N3031<br>• N3041<br>• N3081<br>• N3091<br>• N340<br>• N341<br>• N342<br>• N343	**ICD-10 diagnosis**<br>• N35014<br>• N35028<br>• N351<br>• N358<br>• N359<br>• N360<br>• N361<br>• N362<br>• N365<br>• N368<br>• N99110<br>• N37<br>• N390	**CPT code**<br>• 51702<br>• 51703<br>**ICD-10 procedure**<br>• 0T9B70Z<br>• 0T9B80Z<br>• 0T2BX0Z<br>• 3C1ZX8Z<br>**ICD-10 diagnosis**<br>• B0870<br>• B0871<br>• B0872<br>• N1330<br>• N1339<br>• N139<br>• N200<br>• N201<br>• N202<br>• N209<br>• N312<br>• N319<br>• N320<br>• N3289	**ICD-10 diagnosis**<br>• N329<br>• N3644<br>• N400<br>• N401<br>• N402<br>• N403<br>• N4283<br>• N99510<br>• N99511<br>• N99512<br>• N99518<br>• Q6210<br>• Q6211<br>• Q6212<br>• Q6231<br>• Q6239<br>• R338<br>• R339<br>• R3914<br>• Z436<br>• Z466

Supplemental Table S2. Distribution of Diagnoses in 30-Day Post Index-cUTI period

Total individuals	*n*	(N=723,324) (%)
HIV/AIDS	2,961	(0.4%)
Septicemia, Sepsis, Systemic Inflammatory Response Syndrome/Shock	132,711	(18.3%)
Opportunistic Infections	7,143	(1.0%)
Metastatic Cancer and Acute Leukemia	31,018	(4.3%)
Lung and Other Severe Cancers	17,074	(2.4%)
Lymphoma and Other Cancers	17,094	(2.4%)
Colorectal, Bladder, and Other Cancers	46,105	(6.4%)
Breast, Prostate, and Other Cancers and Tumors	66,750	(9.2%)
Diabetes with Acute Complications	9,421	(1.3%)
Diabetes with Chronic Complications	187,485	(25.9%)
Diabetes without Complication	92,486	(12.8%)
Protein-Calorie Malnutrition	53,653	(7.4%)
Morbid Obesity	56,769	(7.8%)
Other Significant Endocrine and Metabolic Disorders	65,044	(9.0%)
End-Stage Liver Disease	7,859	(1.1%)
Cirrhosis of Liver	8,093	(1.1%)
Chronic Hepatitis	6,016	(0.8%)
Intestinal Obstruction/Perforation	37,244	(5.1%)
Chronic Pancreatitis	4,459	(0.6%)
Inflammatory Bowel Disease	12,977	(1.8%)
Bone/Joint/Muscle Infections/Necrosis	18,053	(2.5%)
Rheumatoid Arthritis and Inflammatory Connective Tissue Disease	72,728	(10.1%)
Severe Hematological Disorders	9,708	(1.3%)
Disorders of Immunity	31,960	(4.4%)
Coagulation Defects and Other Specified Hematological Disorders	90,310	(12.5%)
Dementia with Complications	26,956	(3.7%)
Dementia without Complications	83,178	(11.5%)
Drug/Alcohol Psychosis	2,415	(0.3%)
Drug/Alcohol Dependence	30,242	(4.2%)
Substance Use Disorder, Mild, Except Alcohol and Cannabis	4,816	(0.7%)
Schizophrenia	10,062	(1.4%)
Reactive & Unspecified Psychosis	7,480	(1.0%)
Major Depressive, Bipolar and Paranoid Disorders	79,172	(10.9%)
Personality Disorders	3,484	(0.5%)
Quadriplegia	10,600	(1.5%)
Paraplegia	9,595	(1.3%)
Spinal Cord Disorders/Injuries	14,263	(2.0%)
Amyotrophic Lateral Sclerosis and Other Motor Neuron Disease	988	(0.1%)

Cerebral Palsy	4,708	(0.7%)
Myasthenia Gravis/Myoneural Disorders and Guillain-Barre Syndrome/Inflammatory and Toxic Neuropathy	13,408	(1.9%)
Muscular Dystrophy	755	(0.1%)
Multiple Sclerosis	10,550	(1.5%)
Parkinson's and Huntington's Diseases	25,455	(3.5%)
Seizure Disorders and Convulsions	39,142	(5.4%)
Coma, Brain Compression/Anoxic Damage	13,764	(1.9%)
Respirator Dependence/Tracheostomy Status	10,422	(1.4%)
Respiratory Arrest	1,149	(0.2%)
Cardio-Respiratory Failure and Shock	77,956	(10.8%)
Congestive Heart Failure	179,585	(24.8%)
Acute Myocardial Infarction	39,662	(5.5%)
Unstable Angina and Other Acute Ischemic Heart Disease	22,686	(3.1%)
Angina Pectoris	35,272	(4.9%)
Specified Heart Arrhythmias	200,821	(27.8%)
Cerebral Hemorrhage	14,324	(2.0%)
Ischemic or Unspecified Stroke	54,014	(7.5%)
Hemiplegia/Hemiparesis	27,744	(3.8%)
Monoplegia, Other Paralytic Syndromes	2,309	(0.3%)
Atherosclerosis of the Extremities with Ulceration or Gangrene	11,749	(1.6%)
Vascular Disease with Complications	37,128	(5.1%)
Vascular Disease	198,689	(27.5%)
Cystic Fibrosis	202	(0.0%)
Chronic Obstructive Pulmonary Disease	161,161	(22.3%)
Fibrosis of Lung and Other Chronic Lung Disorders	16,150	(2.2%)
Aspiration and Specified Bacterial Pneumonias	29,447	(4.1%)
Pneumococcal Pneumonia, Empyema, Lung Abscess	23,252	(3.2%)
Proliferative Diabetic Retinopathy and Vitreous Hemorrhage	8,444	(1.2%)
Exudative Macular Degeneration	17,995	(2.5%)
Dialysis Status	16,283	(2.3%)
Acute Renal Failure	168,405	(23.3%)
Chronic Kidney Disease (Stage 5)	3,834	(0.5%)
Chronic Kidney Disease, Severe (Stage 4)	8,238	(1.1%)
Chronic Kidney Disease, Severe (Stage 3)	50,160	(6.9%)
Pressure Ulcer of Skin with Necrosis Through to Muscle, Tendon, or Bone	5,874	(0.8%)
Pressure Ulcer of Skin with Full Thickness Skin Loss	13,399	(1.9%)
Pressure Ulcer of Skin with Partial Thickness Skin Loss	11,864	(1.6%)
Chronic Ulcer of Skin, Except Pressure	12,253	(1.7%)
Severe Skin Burn or Condition	318	(0.0%)
Severe Head Injury	244	(0.0%)
Major Head Injury	12,766	(1.8%)
Vertebral Fractures without Spinal Cord Injury	20,420	(2.8%)

Hip Fracture/Dislocation	23,298	(3.2%)
Traumatic Amputations and Complications	5,897	(0.8%)
Complications of Specified Implanted Device or Graft	58,500	(8.1%)
Major Organ Transplant or Replacement Status	4,453	(0.6%)
Artificial Openings for Feeding or Elimination	31,363	(4.3%)
Amputation Status, Lower Limb/Amputation Complications	1,552	(0.2%)
